# Treatment for problematic substance use in Nordic youth: a narrative review from the viewpoint of social services

**DOI:** 10.1186/s13011-023-00580-9

**Published:** 2023-11-24

**Authors:** Janika Kosonen, Katja Kuusisto

**Affiliations:** Unit of Welfare Sciences, Tampere University, Kalevantie 5, 33100 Tampere, Finland

**Keywords:** Youth, Adolescence, Substance use, Drugs, Alcohol, Drug use, Social services, Child welfare services, Substance use services

## Abstract

**Background:**

Youth mortality from drugs is worryingly increasing in Europe. Little is so far known about what substance use services are available to young people. An out-of-home care placement is often used but does not suffice alone as an intervention in problematic substance use among youth. Additional interventions are needed.

**Objective:**

This narrative review investigated what has been done, what works, and what is needed in treating youth substance use in the Nordic countries from the viewpoint of social services. This study brought together previous Nordic studies on this topic and presented responses to youth substance use in Nordic social welfare system to the wider international audience.

**Methods:**

A search of the ProQuest and EBSCOhost databases revealed seven interventions reported in 17 papers. Narrative synthesis was used.

**Results:**

Interventions included the Cannabis Cessation Program (CCP), the Icelandic version of the Motivation to Change Inventory for Adolescents, the Norwegian multisystemic therapy program (MST), the Structured Interview Manual UngDOK implemented in the Swedish Maria clinics, the Finnish ADSUME-based intervention in school health care, and the Swedish Comet 12–18 and ParentStep 13–17 programs. Many interventions had originated in the US rather than in the Nordic countries and most of them were adapted from adult interventions when youth specificity was lacking. Parental involvement was deemed important, but ineffective without involving the adolescent themself. Interventions and ways for dealing with young offenders required reconsideration from the perspective of the best interests of the child. The current research focuses on universal prevention while more knowledge about selective and indicative prevention was called for.

**Conclusions:**

Not enough is known about the cessation of problematic youth substance use and subsequent rehabilitation in social services. We would encourage further research on the multi-producer system, subscriber-provider-cooperation in youth substance use services, non-medical youth-specific substance use interventions in social services, and rehabilitative juvenile drug offense practices.

## Introduction

Research on under-aged youth and problematic substance use in social services mostly focuses on universal prevention, addressing questions of how to prevent minors from experimenting with alcohol or drugs or how to delay the onset of this behavior. This is reasonable, since the first experiments with alcohol and/or drugs tend to occur during adolescence. The later the onset occurs, the less likely young people are to develop substance use disorders in adulthood [1*, 2, 3]. Internationally, young people are the group most vulnerable to using drugs [4]. However, less is known about how to support young people who have commenced substance use and are experiencing difficulties. An excessive focus on universal prevention may prevent help from reaching young people already dealing with problematic substance use.

Although youth substance use is an increasing concern internationally, the premise of this narrative literature review builds on the prevailing situation in Finland. According to EMCDDA [5], drug mortality among young people in Finland is the highest in Europe. The Finnish national supervisor of social and health care (Valvira) has reported a gap in substance use and mental health care services for minors. They noted that adolescents experience difficulties in obtaining services to which they are legally entitled [6]. The Finnish Safety Investigation Authority (Otkes), in response to a rapid and ongoing increase in drug deaths among young people, recently initiated an investigation into drug mortality among young people under the age of 25 [7]. The latest government program likewise recognizes youth substance use as a phenomenon requiring special measures [8].

The concern in Finland is mirrored in the Nordic countries, i.e., Finland, Denmark, Iceland, Norway, and Sweden. The Nordic countries share a similar societal structure, including an extensive public sector and a comprehensive welfare society, i.e., the Nordic welfare model. In the Nordic countries, the political emphasis of social welfare is on preventive measures [9]. Politically guiding documents such as Sweden’s Comprehensive Strategy for Alcohol, Narcotics, Doping, and Tobacco or Finland’s National Substance use and Addiction Strategy have a strong emphasis on universal prevention of youth substance use [10, 11]. Child Welfare Services (CWS) are required to intervene in cases where the child’s need for care, circumstances, or their own behavior endanger the child’s development. Placement in out-of-home care (OHC) is only feasible after in-home services have proven insufficient [9]. In case of hazardous use of substances, a solution is often sought from an OHC placement. However, it has emerged that the Nordic countries continue to have high numbers of OHC placements together with relatively poor outcomes among care leavers [9]. This would suggest that OHC placement may not adequately meet young people’s needs.

The risk of substance use in adolescence is polarizing. The number of young people who do not use drugs or alcohol has increased, whereas among others the risk of developing a substance use problem has increased [12]. In Denmark, those receiving substance use treatment services are younger and the number of young people using primarily cannabis and cocaine has doubled since 2007 [13]. Internationally, the age of onset for alcohol is estimated to be 16–19, and for cannabis 18–19 and cocaine 21–24 [2]. For comparison, in Finland, the general onset of substance use occurs between the ages of 10 and 15, and amphetamine onset tends to occur between ages 14–17 [12, 14]. Likewise, there is a peak in the number of emergency placements and taking into care for the first time between the ages of 13 and 17 among Finnish youth [15].

Due to the lack of adequate services targeted to youth [6, 7], increasing youth substance use [16–18], and easier access to illicit substances [19], particular attention should be paid to the means to respond to these challenges. In this narrative literature review we examine studies conducted in the Nordic countries and ask 1) *what is known about treatment, programs, or interventions in social services targeted at young people with problematic substance use* and 2) *what does the research in relation to service delivery and interventions recommend?*

## Material and methods

### Data collection

**Fig. 1:**
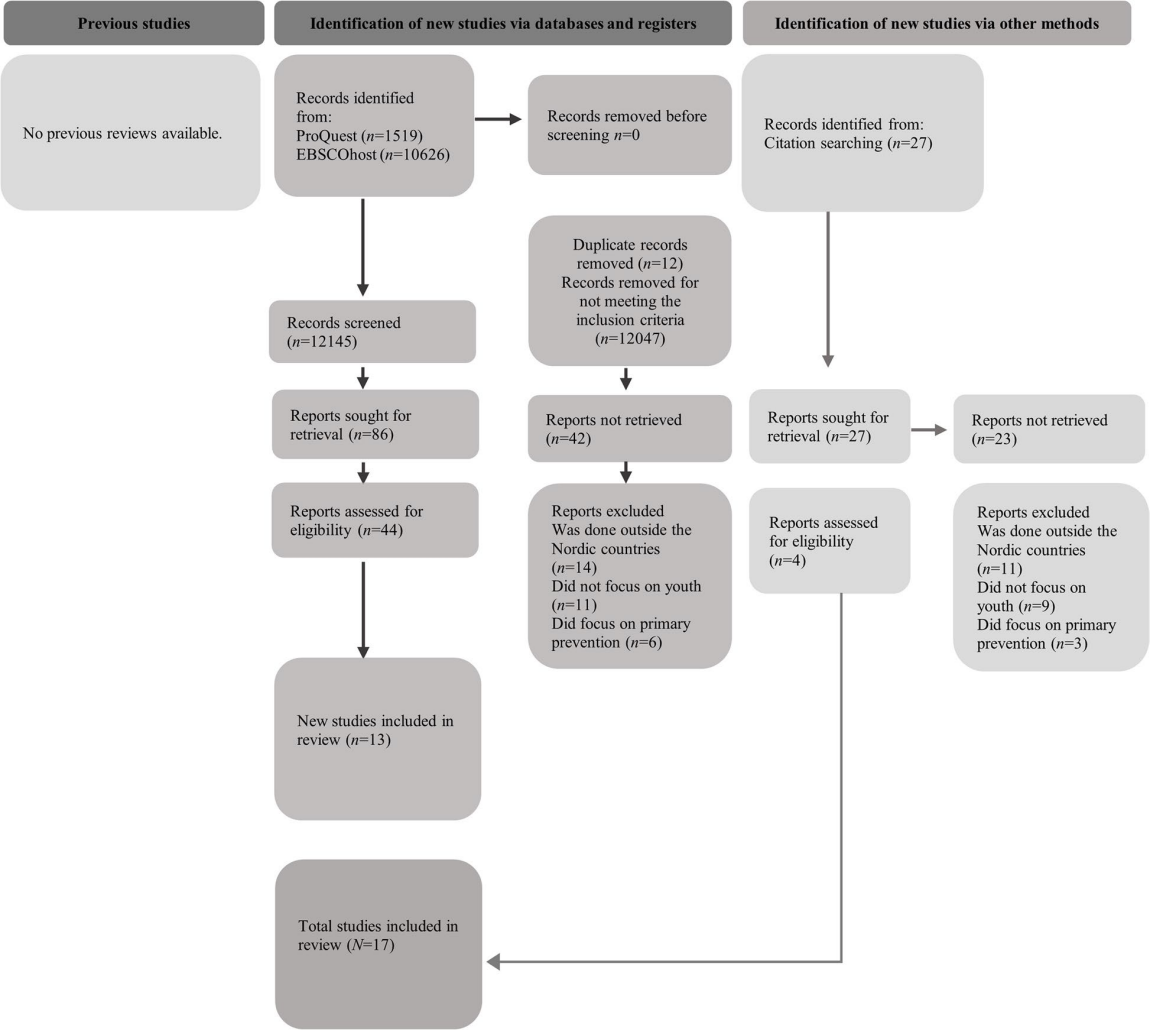
Data collection process for the review of substance use services for youth in Nordic countries

This narrative review follows the PRISMA 2020 guidelines [20]. First, different experimental searches were conducted in various databases. The data collection process is presented in Fig. 1. The following search terms were used: *adolescent OR youth OR minors OR child OR children OR teenagers* AND “*substance abuse” OR “substance use” OR drug OR substance* OR *addiction* AND *service OR services* OR *program* OR *intervention OR interventions* AND *Nordic OR Scandinavia* OR *Finland* OR *Sweden* OR *Denmark* OR *Norway* OR *Iceland* cut to word stem.

**Fig. 2:**
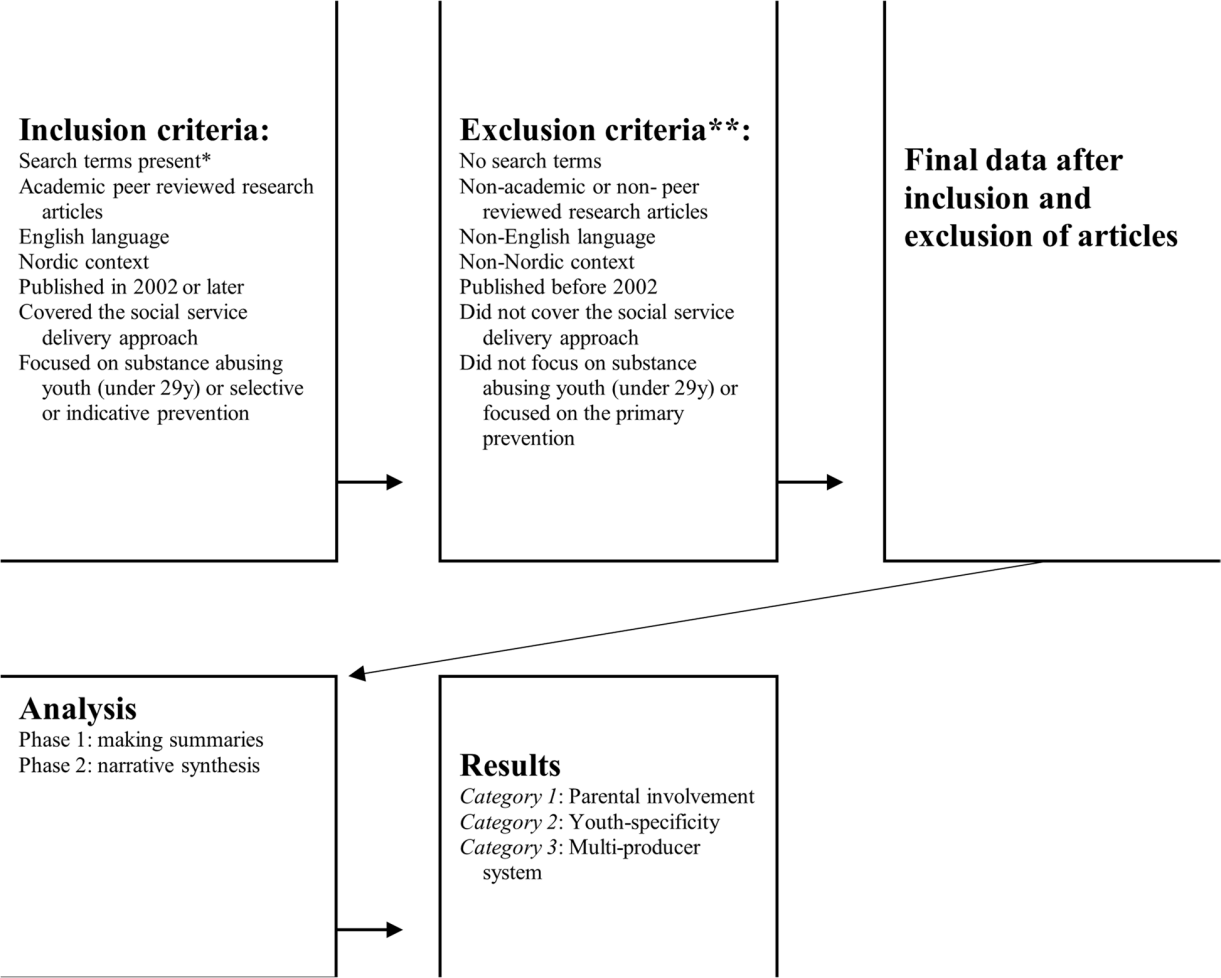
Conduct of research procedure. * Search terms were cut to word stem. ** Evaluated from title, keywords, and abstract

EBSCOhost and Social Services Abstracts at ProQuest databases were used. Following the formation of search terms and test searches in the databases, we created the first version of inclusion and exclusion criteria (See [21]). The criteria were refined multiple times. The final inclusion and exclusion criteria are provided in Fig. 2. The quality of the articles selected was ensured by including only academic peer reviewed articles. The target group of this study was delimited to young people under the age of 29 corresponding to the definition of *a young person* in the Finnish Youth Act (1285/2016). Eighty-six peer reviewed articles were selected for closer examination.

### Method of analysis

All 12047 articles were screened, and duplicates removed, which resulted in 86  articles (see Fig. 1.). Next, all non-Nordic studies and articles that did not cover the social services perspective were removed, which left us with 44 articles. Medical, legal/criminological, and pedagogical articles were excluded. Eventually, articles that did not discuss social services delivery, but only presented correlations, were removed. Articles focusing on universal prevention were likewise removed. Finally, 13 peer reviewed articles were included in the data. Additionally, four articles were included in the data as a result of citation searching. The final data consisted of *N* = 17 peer reviewed research articles.

The analysis followed the logic of narrative synthesis: starting by creating logical categories, then analyzing the study findings within each category, and finally carrying out the synthesis of all studies included [22, 23]. ATLAS.ti program version 22.2.3 was used for data management. Seven intervention models or programs were identified from the data, presented in Table 1.

**Table 1:** Intervention programs and models

Article(s)	Intervention	Target group	Key principles and methods	Duration	Conducted by
Vederhus et al. 2022 [24*]	Cannabis Cessation Program (CCP)	Low-threshold free of charge service for anyone aiming to quit cannabis use	• Manual-based • MI* • Cognitive therapy • Psychoeducative measures • Reflection	~ 15 appointments in 8 weeks	Multidisciplinary staff, e.g., psychologists, nurses, and social workers
Fridjonsdottir 2008 [25*]	Icelandic version of the Motivation to Change Inventory for Adolescents	In this study: Adolescents 14–19 y being admitted for detoxification and/or treatment at Vogur Hospital SÁÁ	• Multidimensional motivation assessment instrument • Adolescent-specificity	Cannot be determined	Hospital staff
Ogden et al. 2008; [26*] Gaulen & Carlsen 2016 [27*]	Norwegian multisystemic therapy program (MST)	Adolescents with anti-social behavior incl. problematic substance use	• Systemized treatment • Multidisciplinary • Referral to the program from municipal CWS*	Cannot be determined	Multiprofessional staff Assessment by regional MST supervisor
Anderberg & Dahlberg 2016; [1*] Anderberg & Dahlberg 2018; [28*] Anderberg et al. 2022; [29*] Boson et al. 2022 [30*] Richert et al. 2020; [31]	Structured interview manual UngDOK implemented in the Swedish Maria clinics	Young people 13–21 y entering treatment in Maria clinics	• Structured interview incl. 75 structured questions • Used systematically with all young people entering treatment • Adolescent-specificity	Treatment in Maria clinic lasts for ~ 4–6 months	Multiprofessional staff at the surface of social services and health care
Pirskanen et al. 2007 [32*]	Finnish ADSUME-based intervention in school health care	Whole age cohorts and the whole range of youth substance use patterns	• ADSUME • Primary, secondary, and tertiary prevention • ADSUME score assists in determining the level of concern and the nature of intervention needed	Cannot be determined	Public health nurses (PHN) at school
Jalling et al. 2016	Comet 12–18	Parents/caregivers and their children aged 12–18 y who were at risk of consolidating antisocial behavior	• Operant learning • Social learning • Video vignettes	9 weeks	Certified social workers working in the family social services
Jalling et al. 2016	ParentStep 13–17	Parents/caregivers and their children aged 13–17 y who were at risk of consolidating antisocial behavior	• Resilience Model (Richardson et al. 1990) • Social Ecology Model of Adolescent Substance Use (Kumpfer & Turner 1990) • Video vignettes • Parent sessions only	6 weeks	Certified social workers working in the family social services

During the analysis an overall picture of the data was formed. At this stage, individual observations were combined into broader categories (see [22, 23]). The observations were formed into categories due to consistency, repetition, and/or general significance in relation to the research questions.

Eventually, three logical categories were created: 1) parental involvement, 2) youth-specificity, and 3) multi-producer system. After forming the categories, each category was internally analyzed by observing similarities and differences. The results of the analysis are presented by category. The conclusive synthesis is performed in the discussion.

## Results

The final data consisted of *N* = 17 peer reviewed articles published between 2007 and 2022 (see Table 2). Six articles were qualitative studies [26*, 34, 27*, 32*, 33*, 35*], whereas 11were quantitative  [1*, 31, 24*, 25*, 28*, 29*, 30*, 36*, 37*, 38*, 39*]. Most of the articles were Swedish (*n*=8) and Norwegian (*n* = 6), and the rest were Finnish (*n* = 1), Danish (*n* = 1), and Icelandic (*n* = 1).

**Table 2:** Data information

**Research/result category**	**Layout**	**Data**	**Aim**	**Key findings**
Anderberg & Dahlberg & Wennberg **2022, Sweden **[29*] **2, 3 **	Quantitative follow-up study	Combined data from structured interviews with young people at intake and data from various registers at follow-up 1 year after baseline. *N* = 455	To analyze indications of continued problems with criminality and drug use among young people, and how various risk factors predict outcomes 1 year after initiated treatment contact at outpatient clinics	About one-quarter of the young people who begun outpatient treatment had been convicted of crimes at 1-year follow-up. Most of them who had been convicted of offenses also had ongoing problems with substance use and three-quarters of the young people had been charged with a drug-related offense. Interventions meeting the needs of young people and moving away from the emphasis on penal law principles were recommended
Anderberg & Dahlberg **2018, Sweden** [28*] **1, 2**	Cross-sectional quantitative study	Data from structured UngDok interviews from 11 Swedish Maria clinics. *N* = 2169	To describe similarities and differences regarding various risk factors between girls and boys with substance abuse problems who begin outpatient treatment	Girls appeared to have more difficult family and childhood environments than boys and were more likely to have problems related to school, more serious substance abuse problems, and more severe mental health problems. Criminal activity was significantly higher among boys
Anderberg & Dahlberg **2016, Sweden** [1*] **1, 2**	Quantitative study	UngDOK intake data from Maria clinics. *N* = 748	To describe and analyze victimization among adolescents who are in outpatient treatment for substance abuse disorders with respect to gender, social circumstances, alcohol and drug abuse, and mental health	More than half of the adolescents had experienced violence or other type of abuse. There were significant gender differences: two thirds of the girls and slightly less than half the boys had experienced abuse in some form, and the girls had more severe needs at treatment admission
Boson & Anderberg & Hagborg Melander & Wennberg & Dahlberg **2022, Sweden **[30*] **2, 3**	Quantitative follow-up study	Data from official records of Maria clinics from 12 medium-sized to large cities. Combined data from structured interviews with adolescents at intake and data from various records at follow- up 1 year after baseline. *N* = 455	To describe and analyze indications of mental health problems and how various risk factors predict outcomes 1 year after initial treatment contact. Links between risk factors at the individual, social, and structural levels as well as links between various mental illness symptoms at treatment start and potential indications of mental health problems 1 year later were analyzed	Mental health problems among adolescents largely persisted 1 year after starting outpatient care for substance use problems. Forty-two per cent of the sample had mental health problems at follow-up, and registrations for both outpatient treatment and psychiatric medication were more common among girls. Girls reported more mental illness symptoms at treatment start, especially anxiety
Ekendahl & Månsson & Karlsson **2020, Sweden **[33*] **3**	Qualitative narrative/thematic study	Semi-structured interviews for 16–24 years old young clients at six outpatient treatment centers for young people with substance abuse problems. *N* = 18	To explore how young people perceive outpatient treatment for cannabis use, position themselves as subjects in relation to it, and how they respond to staff’s appeals to rationality and responsible action	Young clients understood their histories in a responsibilized way where the risk information about cannabis they received was considered crucial. Those who resisted treatment rejected cannabis problematizations by staff, did not value interventions and felt that they managed their use. Those who complied with treatment said that cannabis problematizations helped them acknowledge their own difficulties, handle addiction and mature
Fridjonsdottir **2008, Iceland** [25*] **2**	Quantitative study	Records of 14–19 years old adolescents admitted for detoxification treatment at the Iceland’s main detoxification and treatment center. *N* = 41	To explore if the *Motivation for Change Inventory for Adolescents* (Revised)-Icelandic version is valid and reliable instrument for measuring motivation for change in the early phase of chemical dependency treatment among adolescents	Fourteen items in the *Motivation to Change Inventory of Adolescents* (Revised)-Icelandic version have acceptable internal consistency reliability. These items measure self-efficacy, social support, alternative activities, and perception of life skills; these variables are all related to motivation for change, a multidimensional construct
Gaulen & Carlsen **2016, Norway **[27*] **2, 3**	Descriptive qualitative review	Contains no empirical data	To give an overview about the current youth substance abuse recovery services and intervention practices in Norway, and to describe available opportunities for and barriers to youth treatment and aftercare	Norway has several adolescent substance abuse treatment centers and services in Norway. The responsibility to help youth who need alcohol and/or drug treatment lies in the Norwegian municipalities. Treatments take place in institutions or at outpatient clinics. Young people typically receive detoxification services in a hospital or residential treatment setting
Heradstveit & Gjertsen & Iversen & Aasen Nilsen & Gärtner Askeland & Christiansen & Hysing **2020, Norway **[36*] **3**	Quantitative comparative study	Cross-sectional, population-based survey data. *N* = 9785	To investigate whether adolescents in contact with CWS are at higher risk for substance-related problems (SRP) compared with the general adolescent population, and to what extent those in foster care (FC) differ from those receiving in-home services (IHS)	Adolescents receiving IHS and adolescents in FC had a significantly heightened risk for SRP, compared with the general population. The risk for SRP was higher among adolescents receiving IHS compared with those living in FC
Jalling & Bodin & Romelsjö & Källmén & Durbeej & Tengström **2016, Sweden **[37*] **1**	Quantitative simple randomized trial	Records of parents and their adolescents aged 12–18 who were assigned either to a Comet 12–18 or a Swedish ParentStep program due to the adolescent being at risk for consolidating antisocial behavior. *N*_parents_ = 241; *N*_adolescents_ = 237	To investigate the effects of Comet 12–18 and ParentSteps on measures of antisocial behavior when given under real-world conditions to parents of at-risk adolescents	Neither Comet nor ParentSteps had beneficial effects on adolescents’ antisocial or delinquent behavior, or on alcohol use. The risk of illicit drug use at follow-up was threefold greater in both intervention groups compared to controls, suggesting that the studied parenting programs might have caused harm in this particular outcome. However, this finding should be treated with caution for several reasons
Järvinen & Ravn **2015, Denmark **[34] **1**	Qualitative narrative study	Semi-structured qualitative interviews with young people who enrolled in outpatient drug treatment. *N* = 30	To show that images of the past are related to images of the future: the way the interviewees narrate their cannabis career up until now is reflected in the way they anticipate their relationship to drugs in the years to come	Four different drug use ‘etiologies’ were identified drawn upon by the interviewees. These included childhood experiences, self-medication, the influence of friends and cannabis use as a specific lifestyle
Nordfjærn & Dahl & Flemmen **2013, Norway **[38*] **1, 2**	Quantitative study	A cross-sectional questionnaire survey data. *N* = 1288 (*n* = 740 nine secondary schools and *n* = 548 three high schools)	To investigate social influence, health, criminality and substance use among Norwegian rural adolescents. Relations between these factors and substance use were examined	Deviant behaviors had higher social status among males and adolescents in high school. The social status of deviant behaviors and participation in criminal activities were associated with alcohol and illicit substance use. Parent–adolescent trust was positively associated with alcohol use and parent involvement with friends was similarly related to illicit substance use. The social status of physical appearance and talent in sports were negatively associated with alcohol use
Ogden & Christensen & Sheidow & Holth **2008, Norway **[26*] **3**	Descriptive qualitative review	Contains no empirical data	To describe the successful nationwide transport and evaluation of Multisystemic Therapy (MST) programs in Norway	Norwegian governmental policy reforms to improve services for young people and their families have focused on evidence-based treatments that take a family preservation approach. The successful nationwide transport and evaluation of MST programs in Norway has much application to US efforts for large-scale adoption and implementation of evidence-based practices. MST Norway has effectively addressed barriers at both the system level and practitioner level
Pirskanen & Laukkanen & Pietilä **2007, Finland **[32*] **1**	Qualitative study	School health nurses (*n* = 510) tested ADSUME-instrument and early intervention on 14–18 year-old adolescents (*n* = 5228). Six months later, the nurses and their professional partners (*n* = 24) were invited to assess EI in focus group interviews	To improve an early intervention (EI) triggered by the Adolescents’ Substance Use Measurement (ADSUME) as a method to prevent substance abuse among adolescents	ADSUME concretized assessment, activated profound dialogue, and proved to be an important part of EI. It was important to assess the adolescent’s resources in addition to the ADSUME score. EI worked well in confidential dialogues after the adolescent and the PHN reached a consensus on the level of concern about the adolescent’s substance use. The recommended EI enabled individual brief intervention in all four stages of substance use, from abstinence or experimental use to hazardous use
Richert & Anderberg & Dahlberg **2020, Sweden **[31] **2, 3**	Quantitative study	Structured interviews with* N* = 1970 young people enrolled in Maria clinics in 11 Swedish cities	To analyze self-reported mental health problems among young people receiving outpatient treatment for substance use problems	Self-reported mental health problems were common among the young people. A relatively large percentage of the total group (34–54%) reported problems such as concentration difficulties, sleeping difficulties, anxiety and depression. At the same time, many of the young people did not report any symptoms and only a small group reported diagnosed mental health disorders. The results show substantial gender differences, with girls reporting significantly higher levels of mental health problems. Multivariate logistic regression analyses demonstrated significant associations between severity of drug use problems and anxiety, concentration difficulties, aggression, hallucinations and mental stress caused by experiences of trauma
Sandøy** 2019, Norway** [35*] **1, 3**	Qualitative study	Semi-structured interviews with *N* = 22 young (15–17 y) offenders in four different locations in Norway	Based on in-depth interviews with youth enrolled in programs to help them refrain from drug use, to identify how the early-stage desistance process is understood by would-be desisters	Rather than describing the rehabilitative programs’ direct impact on their behavior and thinking, the adolescents emphasized the importance of restoring relationships with parents and overcoming legal obstacles. Concerns with personal reform were outweighed by (i) social and (ii) legal concerns. While the precedence of external concerns over personal reform may reflect the participants’ age and level of criminal involvement, it also reflects a particular culture of intervention
Vederhus & Rørendal & Skårdal & Næss & Clausen & Kristensen & López-Goñi** 2022, Norway **[24*] **2**	Quantitative study	The respondents (*N* = 102) were recruited in four community-based Cannabis Cessation Programs (CCP) in Norway	To examine outcomes of the Cannabis Cessation Program (CCP) intervention	Seventy-six participants (75%) completed the 8-week program, according to plan. All participants reported a significant reduction in cannabis use at T1 (average reduction ~ 16 days per month) and at T2 (N = 59; ~ 13 days per month). Among those that completed the pro- gram, 67% was abstinent from cannabis at T1 and 37% was abstinent at T2. An intention-to-treat analysis showed that 50% (51/102) and 22% (22/102) were abstinent from cannabis use at T1 and T2, respectively. In parallel to abstinence, substantial reduction in mental distress and an increase in well-being and sense of coherence (SoC) were observed. Respondents socialized with fewer friends with current substance use, but drug-free social networks were not expanded
Åström & Jegerby & Andershed & Tengström **2013, Sweden **[39*] **1**	Quantitative vignette study	Vignette-based questionnaire data. The respondents were social workers employed in social services in Stockholm’s area	To examine how social workers assess adolescents with substance misuse problems, criminal behavior and mental health difficulties, and how they make decisions about treatment interventions to reduce these problems	Social workers recognized the problems and needs of young people, but that they found it harder to link needs to evidence-based interventions

A narrative synthesis of the findings yielded three themes, namely 1) parental involvement, 2) youth specificity, and 3) multi-producer system.

1. Parental involvement

We found seven studies ([1*, 34, 28*, 32*, 35*, 37*, 38*, 39*], see Table 2) that discussed parental involvement in intervening in young people’s problematic substance use.

In Sweden, it was found that interventions targeting only the parents of at-risk adolescents were not effective in intervening a young person’s problematic substance use. In fact, the study found that parent training interventions targeting only the parents might even have increased the risk of illicit substance use among adolescents [37*]. However, parental involvement together with the involvement of the adolescent themself had been found to be essential when intervening in problematic youth substance use both for the intervention to be successful, and for the young people themselves [32*, 35*]. According to Pirskanen and co-workers [32*], involving parents in the adolescent’s treatment process helped the professional to identify existing protective and risk factors and thus provide the right kind of support. In addition, to this, Sandøy [35*] found that young people participating in a Norwegian offender management program for drug offenses seemed to place particular emphasis on building relationships with their parents, which promoted positive change. In contrast to these studies, Nordfjærn and co-workers [38*] discovered that parental involvement and a trusting parent-adolescent relationship did not always mean positive outcomes in terms of problematic youth substance use.

According to Anderberg and Dahlberg [1*, 28*] girls with problematic substance use seemed to receive less support from their own parents than did boys with similar issues. In fact, boys entered the care system more often at their parents’ instigation [28*]. Girls seemed to have experienced more difficult family environments in their childhood than boys. Järvinen and Ravn [34] found that young people in cannabis cessation treatment attributing their problematic cannabis use to adverse childhood experiences seemed to find the management of their drug problem more challenging. They tended to adopt a fatalist approach to their situation.

Jalling and co-workers [37*] evinced one possible explanation for why parent-only interventions do not work with young people; it might be that they had often already distanced themselves from their parents and therefore tended to spend more time outside the home. The role of the peer relationships was highlighted, and peers had taken over the place of the parents. According to this study, the older the young people were, the less influence their parents seemed to have over their behavior and actions. However, Sandøy [35*] found that for young people with a history of drug offenses, parents seemed to represent so-called informal social control, which may be conducive to the cessation of substance use. This appeared in the young people’s narratives, where desistance from crime and/or substance use was not seen as an objective in itself but was understood as a tool to restore the trustful relationship with the parents. In their vignette study, Åström and co-workers [39*] found that Swedish social workers proposed counseling as an intervention for adolescents with problem behavior, including problematic substance use, in preference to evidence-based options and family-oriented interventions. Family-oriented interventions were not advised for adolescents because the vignette cases described a good relationship between the adolescent and the parents. According to the authors, this is comprehensible, although they claimed that also these parents could need support in strengthening their parenting skills which could positively impact the adolescent’s situation. Sandøy [35*] claimed that a good parental relationship can be conducive to the young person’s desistance from substance use through informal social control.

Parents are not always willing to participate in their adolescent’s change process. Pirskanen and colleagues [32*] found that parents were more likely to become involved in their adolescent’s case when the adolescent had other co-occurring concrete problems in addition to substance use. Hence, the parents could be motivated by the urgency to act. In Norwegian rural settings Nordfjærn and colleagues [38*] discovered that parent-adolescent trust and parental involvement in the adolescent’s social relationships might even increase the risk for illicit substance use. This could be a consequence of permissive parental attitudes towards substances. Intervening in the problematic substance use of a young person required the parents to acknowledge the problem, since they seemed to exercise informal social control over their adolescent as a possible means to with which they may influence their behavior and actions [35*]. A trustful adolescent-parent relationship might also indicate that the adolescent experienced more autonomy in general [38*]. Hence, the parents might even blindly trust their child or experience difficulties in believing their child’s substance use.

2. Youth-specificity

Nine studies reviewed youth as a special phase of life. Youth was also seen as a good time for substance use interventions, although according to these studies, specific features related to adolescence included difficulties to commit, impulsive decision-making, short history of substance use, and changes in social circles ([1*, 31, 24*, 25*, 27*, 28*, 29*, 30*, 38*]; see Table 2). Moreover, accessibility of substance use services was inadequate, and interventions targeting young people came often too late [27*].

The younger the participants are, the more common it is to drop out intervention programs and the more challenging it is to proceed persevere with the program structure [24*]. Also, due to a short history of substance use, even in detoxification, young people tend to perceive their own substance use as unproblematic in contrast to their parents’ perceptions [25*]. However, young people frequently accessed substance use services only when the risks had already realized, and the substance use had already become problematic [27*]. Thus, these special features related to adolescence pose unique challenges for substance use services.

Adolescence is also a time of changes in social circles, e.g., detaching from parents, spending more time with friends, and changing schools (e.g., from secondary to high school) [38*]. Järvinen and Ravn [34] found that one narrative which young people used to explain their problematic cannabis use was peer relationships. This was identified as agency-oriented narrative and young people who used this narrative were optimistic with regard to ceasing to use cannabis but understood that it necessitated changes in their social networks. Nordfjærn and colleagues [38*] found that the transition from attending school while still living at childhood home into continuing their school away from home seemed to be a particularly risky time in relation to the development of problematic substance use, especially among rural youth. For these young people the detachment from the parents is usually also physical, since they typically move away from their parents to go to school in bigger cities.

Close attention should be paid to gender differences regarding problematic youth substance use. Several studies have reported that young girls grappling with problematic substance use tend to have more traumatic backgrounds than do young boys dealing with similar issues [1*, 2, 16, 19, 40,28*, 29*, 30*, 33*, ] although history of victimization within this group is common among both binary genders [1]. Anderberg and Dahlberg [28*] showed that girls’ substance use problems seemed more serious than those of boys in terms of more frequent use of drugs and more common polydrug use. Females tend to access the mental health open care services more readily than males and they are more likely to be sentenced to the so-called “youth contract” compared to males in case of drug offenses [29*, 30*]. Boys with problematic substance use show are more likely than girls to commit crimes but Anderberg and Dahlberg [1] found that girls experienced more difficulties in controlling their violent behavior. Girls tend to enter the care system voluntarily or through the health care system, whereas boys are more likely to enter treatment through their parents or social services [28*]. Nordfjærn and colleagues [38*] suggest that early interventions should specifically target young males who seem to experience more difficulties in accessing services.

According to Richert and colleagues [31], almost half of girls and a quarter of boys dealing with problematic substance use had experienced a severe traumatic event from which they had not fully psychologically recovered. Since girls seemed to have experienced more difficult childhood environments and been subjected to more forms of violence, it was suggested that girls might need more comprehensive treatment interventions. Several papers revealed an extensive need for gender-specific knowledge and practices in the context of youth substance use services [29*, 31, 30*, 38*].

3. Multi-producer system

Multi-producer system, including Child Welfare Services (CWS), and juvenile drug offense policy were discussed in eight articles [26*, 30, 31, 27*, 29*, 33*, 36*]. Multi-producer system describes the present service system based on subscriber-producer model where several service subscribers and producers can simultaneously work with the same client. CWS are required to intervene in cases were the child’s environment or own behavior, e.g., problematic substance use, are evaluated as harmful for the child’s healthy growth and development. Due to a rapid privatization, many child welfare and substance use services are acquired through public procurement from private or third-sector service providers, which creates new challenges to act in such multi-producer environments. Social work authorities monitor the best interests of the child in legal processes and are therefore involved in the juvenile drug offense policies.

Findings in the Nordic countries have been inconsistent with those reported elsewhere about young people involved in child welfare services in their own homes being at higher risk of developing substance-related problems than those in foster care [36*]. Foster care, however, has been found to predict persistent mental health problems while the social networks of young people with foster care experiences may be weaker than those without such experiences [30*]. Compared to the population, young people with CWS experiences are at higher risk for developing substance-related problems [36*]. OHC placement is often implemented in case of hazardous youth substance use due to the need for protection and lack of other services. In Norway, the multisystemic therapy (MST) program has proven to be more successful than the habitual CWS measures in reducing placements and problem behavior reported by teachers and parents as well as self-reported criminal activity of adolescents [26*].

Multidisciplinary approaches to intervening in problematic youth substance use were deemed essential [31]. In a such multi-producer system, the importance of cooperation and coordination among services and stakeholders was emphasized [27*]. According to Richert and colleagues [31] in adolescence, the underlying causes for risks to become realized seem to be embedded in social issues, thus medicalization is seen as a potential risk in organizing youth substance use services. Professionals should collaborate at the interface of social and health care while closely and continuously cooperating with service subscribers and producers [27*, 31].

Use and possession of almost all drugs, including cannabis, is illegal in the Nordic countries. However, instead of the traditional legal punishments, alternative penal sanctions were increasingly implemented for juvenile drug offenders [29*, 33*, 35*]. In Sweden, cannabis use mostly results in the young offender being sent for outpatient cannabis cessation treatment [33*]. Alternative sanctions for juvenile drug offenders were recommended by researchers to prevent social exclusion from escalating [29*, 35*]. Concurrent interventions to intervene in both problematic substance use, and delinquent behavior have been recommended as an alternative to traditional penalties [29*]. The authors of the articles reviewed recommended more nuanced interventions to meet individual needs and take account of the social conditions of these young people [29*, 33*, 35*].

Although treatment interventions seemed to be recommended as an alternative to traditional punishments, only little was known about what kinds of interventions work, especially with young people who use substances. According to Gaulen and Carlsen [27*] this might be because prevention focuses chiefly on the stage of universal prevention, with very little attention paid to selective and indicative prevention, i.e., to those young people already using substances or dealing with substance-related problems. Constructing a more holistic juvenile justice system was deemed necessary [27*, 29*].

## Discussion

In this narrative review we examined what kinds of interventions have been implemented in social services to address problematic youth substance use in the Nordic countries. Accordingly, we asked, *what works* and *what is needed*? The research on this aspect is limited. So far, research about youth substance use in social services has focused on universal prevention, yet polarization of substance use, increasing drug mortality among young people, and lack of substance use services were attested to (see e.g., [27*]). More research is needed on the levels of selective and indicative prevention since these young people experience difficulties in accessing the service system. Nordic comparison in this field is scarce, probably because Nordic welfare models are commonly seen as similar to each other in spite of significant differences [41].

Various intervention programs and models have been implemented. Typical components were motivational interview (MI) and various structured interview methods and tests, such as UngDOK, AUDIT, DUDIT, ADSUME, and the Motivation to Change Inventory for Adolescents. Evidence-based intervention programs implemented in Nordic settings were mainly adopted from the US [25*, 26*, 27*]. Parental involvement was deemed essential, but interventions targeting only the parents were deemed unsuccessful [32*, 35*, 37*]. Boys were more likely to enter the care system through their parents whereas girls experienced less parental support [28*].

Adolescence was acknowledged to create specific prerequisites for treatment, and thus it challenges substance use interventions. Still, it was conceded that the knowledge-base mainly originated in adult contexts [24*, 31, 25*, 27*, 29*, 30*, 38*]. Gender differences between the binary genders form another challenge for treatment delivery [28*, 31, 29*, 30*, 38*]. They indicated that girls and boys with problematic substance use required specific support, but for differing reasons. Multidirectional cooperation between all actors at different levels was deemed essential when organizing and planning youth substance use interventions [27*, 31]. Medicalization was identified as a potential risk [31*] as medical solutions continue to expand to new areas. Suggestions were made to reconsider the juvenile drug offense policy and practices from the perspective of the UN Convention on the Rights of the Child [29*, 33*, 35*].

The large numbers of young people placed in OHC indicated that the political ideology of preventative emphasis on both substance use services and CWS was not fulfilled in practice (see e.g., [9]). The existing interventions were criticized for being unable to meet the individual needs and pay attention to social conditions of young people using substances [29*, 33*, 35*]. Highly structured intervention programs and models also lacking such flexibility might exacerbate the issue. Structured, manually based interventions standardize processes and encounters and are therefore suitable for universal prevention. We argue, that for selective and indicative prevention and harm reducing approaches, manually based interventions lack the ability to sufficiently meet the individual needs of young people who use substances, and thus they should not be primarily used for those purposes.

Due to the lack of research in this context, services and interventions have mainly been developed on the basis of information gathered from adults. This is problematic since in adolescence there are specific prerequisites to execute interventions. Also, adolescents’ life situations as well as their rights and responsibilities are often different from those of adults. It has already been conceded that the Nordic welfare model does not perceive children and young people as independent actors, but rather as involved in society as family members [41]. Recognizing young people as independent actors is a prerequisite of meeting their individual needs in the service system.

Social work seems to have an essential place operating at the interface of social welfare and health care together with stakeholders in various sectors. The client-centered coordination of different services and actors is one of the main tasks in social work and due to today’s multi-producer system the need for expertise in this area is increased. To response to the ongoing medicalization in this field of practice, the role and competence of social services must be strengthened. Medicine has an important and solid role in the service system, but medicalization means the expansion of its role to such areas it did not previously belong to.

This narrative review includes some limitations that must be considered. First, only articles written in English were included in the data, which probably excluded several relevant articles written in Nordic languages. Research on this topic referring to social services was limited. Due to the lack of research, the data also included two review articles that did not contain empirical data. They were, however, included since they contained relevant information on various intervention programs and models. Despite the limitations, the present paper successfully scrutinized the scarce Nordic research on the topic, and this is valuable for forthcoming research.

The results of this study have several practical implications for the field of substance use services. Firstly, the results highlight the need for youth-specific substance use services. The results of this study can be utilized when reconsidering and developing these services at the interface of social welfare and health care. Specificity to youth, sensitivity to gender, and trauma-informed orientation are to be promoted. Moreover, this paper addresses the topics of juvenile drug offense policies and practices, thereby also contributing to those discussions. Practitioners working in the fields of social welfare, health care, and juvenile delinquency can benefit from this paper whether working with clients, in development, or in management.

The present study also has several implications for future research. It presents the relevant Nordic research comprehensively and reveals significant research gaps. The phenomenon of youth substance use desistance remains insufficiently known. The specific prerequisites and challenges related to youth substance use interventions should be investigated in more detail. Research and evidence-based interventions have often been adopted to the Nordic countries from the US. However, since the Nordic welfare model is in many ways different from the US model, increased Nordic cooperation is needed.

## Conclusions

Research on problematic substance use among young people is mostly focused on universal prevention when it comes to treatment from the viewpoint of social services. Not enough is known about the cessation of problematic youth substance use and subsequent rehabilitation. In light of the existing research, we would encourage further research on the multi-producer system, subscriber-provider-cooperation in youth substance use services, non-medical youth-specific substance use interventions, and rehabilitative juvenile drug offense practices. Finally, increasing Nordic and international cooperation in developing and implementing interventions for youth is recommended.

## Data Availability

All data generated or analyzed during this study are included in this published article and its supplementary information files.

## Abbreviations

ADSUME: Adolescents’ substance use measurement
AUDIT: Alcohol use disorders identification test
CWS: Child welfare services
DUDIT: Drug use disorders identification test
MI: Motivational interview
OHC: Out-of-home care
